# Evaluation of protein quantity and protein nutritional quality of protein bars with different protein sources

**DOI:** 10.1038/s41598-025-94072-4

**Published:** 2025-03-18

**Authors:** Judit Tormási, Eszter Benes, Éva Lengyel Kónya, Mária Berki, László Abrankó

**Affiliations:** Institute of Food Science and Technology, Department of Food Chemistry and Analysis, Hungarian University of Agriculture and Life Sciences (MATE), Budapest, Hungary

**Keywords:** Protein source, Plant protein, Animal protein, DIAAS, In vitro digestion simulation, Malnutrition, Analytical chemistry

## Abstract

A comprehensive overview of commercially available protein bars, focusing on their protein content, protein source(s) and nutritional composition. Four protein bars were selected based on the quality of their protein sources; (i) plant only (pea and rice); (ii) animal only (milk proteins); (iii) mix of animal (milk and egg) and plant (soy); (iv) mix of animal (milk and collagen) and plant (soy) to assess the relationship between protein sources and protein nutritional quality. Data analysis was conducted on data from an online consumer-generated database (OpenFoodFacts.org). Indeces of protein nutritional quality, DIAAS and PDCAAS, were determined after in vitro digestion simulation using the Infogest method. Of the 1641 bars, 81% had sufficient protein to be classified as “high in protein” (protein content > 20E%). However, the results show that lower protein digestibility values (between 47 and 81%) were measured when the proteins were included as a component of the protein bar matrix than when the digestibility of the same proteins was evaluated in a pure format. All measured in vitro-DIAAS and PDCAAS values were relatively low with the highest DIAAS = 61(Trp) and PDCAAS = 62(Trp) obtained for a protein bar containing only milk proteins (WPC, MPC). Although most protein bars are labelled ‘high in protein’, their protein nutritional quality could be very low based on DIAAS. The low numbers are most probably due to application of lower-nutritional-quality proteins (such as collagen) and of other ingredients such as carbohydrates, fats and fibres, that might deteriorate the bioaccessibility of essential amino acids.

**Supplementary Information:**

The online version contains supplementary material available at 10.1038/s41598-025-94072-4.

## Introduction

Protein bars are a convenient and nutritious form of a protein and energy. Originally developed for athletes but now also targeted to appeal to a wide range of health-conscious consumers^16^. Protein bars usually contain 10–40 g of protein mostly of dairy and soy origin^14^. However recent shift in consumer behaviour encouraged the use of alternative plant-based protein sources such as pea and rice^17^. Although protein bars are known as a “good source of protein” most research are focusing on their techno functional and rheological properties e.g., since the incorporation of protein powders requires a high ratio of additives to create palatable products^14,16,19^. Nonetheless, there is negligible data on their nutritional quality. In addition, the question whether the name “protein bar” could be misleading in some cases have been raised previously^11^. Fernan and colleagues have found that producers include “healthy” sounding and sometimes misleading labelling and claims “*protein source*” or “*high in protein*” not in line with labelling regulations^23^ which might affect consumer perception of protein bars^11^. However, there is no indication of their superiority amongst snack or energy bars based on protein nutritional quality.

Protein nutritional quality could be expressed by two FAO/WHO recommended indicators; the PDCAAS and the DIAAS. For many years, protein quality has been assessed using the PDCAAS from the product’s limiting amino acid and its faecal protein digestibility^15^. This approach is limited by several factors; i.e., (i) digestibility is determined based on nitrogen content of the faecal sample which includes nitrogen deriving from microorganisms in the large intestine, (ii) digestibility of individual amino acids is neglected although it is depending on protein composition, structure and other components of the food matrix, (iii) values have to be truncated above 100% due to methodological limitations. In light of these issues, the Food and Agriculture Organization has proposed a new indicator called DIAAS to better assess protein nutritional quality^10^. DIAAS is proposed to be determined at the ileal level of human or pig small intestine based on the bioavailability of individual amino acids. Recent shift in research ethics created a need for the interpretation of the in vivo methodologies to in vitro protocols. Therefore several studies focused on the creation and validation of an in vitro DIAAS determination method in line with the in vivo protocols^3,8,9^. Finally in 2023, a standardized protocol has been introduced by Sousa and colleagues validated for animal and plant-based protein sources as well^22^.

Nutritional quality of individual protein sources has been long studied. There is a consensus that encourages the consumption of animal-based proteins (i.e., meat, eggs and dairy) over plant-based protein sources (i.e., soy, pea or rice) due to their complete amino acid composition compared to plant proteins’ incompleteness^13,20^. The main source of protein in many protein bars are dairy originated proteins such as whey protein concentrate/isolate (WPC/WPI) or milk protein concentrate/isolate (MPC/MPI). However due to change in consumer behaviour plant protein sources are often used as an addition to animal protein sources or by themselves, as a mix of plant-based proteins^17^. Although dairy and other animal-based proteins count as good sources of protein, protein bars usually contain additional components as chocolate, sugars, and flavourings to create an appealing taste and flavour, nuts, wafers, nuggets for texture, and added vitamins, minerals, and/or fibre for enhanced nutritional value^16^. These additional components all affect the digestibility of proteins and accessibility of amino acids therefore could considerably change their protein nutritional quality^6^.

In this work, we used data from a consumer-generated database to establish the relationship between the protein nutritional quality and the source of protein in protein bars. First, the applicability of protein claims was assessed and the association with other nutrients (carbohydrates, fats and fibre) was established. Next, relevant protein sources were identified and bars were categorised according to their primary and secondary protein sources. Based on these results, four protein bars - containing the most relevant protein source pairings - were selected and evaluated for their DIAAS after in vitro digestion simulation. We believe that this research addresses a much neglected aspect of protein bars, i.e. that high protein content does not necessarily mean ‘high’ protein nutritional quality, and provides gap-filling information as a basis for further research.

## Results

### Protein content of protein bars

Our data was selected from the website OpenFoodFacts.org using the keyword “protein bar”. The original 4638 protein bars found by the search engine were curated based on several criteria thus protein evaluation was made on only 1669 bars. From the 1669 protein bars 1641 had available information on nutritional composition to calculate ratio of protein content, thus 28 bars (without protein content information) were excluded due to the lack of protein content and/or ingredient list. The studied 1641 bars could be classified as follows according to EU labelling criteria on protein related claims: “not protein source” 18 bars (1%), “protein source” 295 bars (18%), and “high in protein” 1328 bars (81%).

### Protein sources of protein bars

**Fig. 1:**
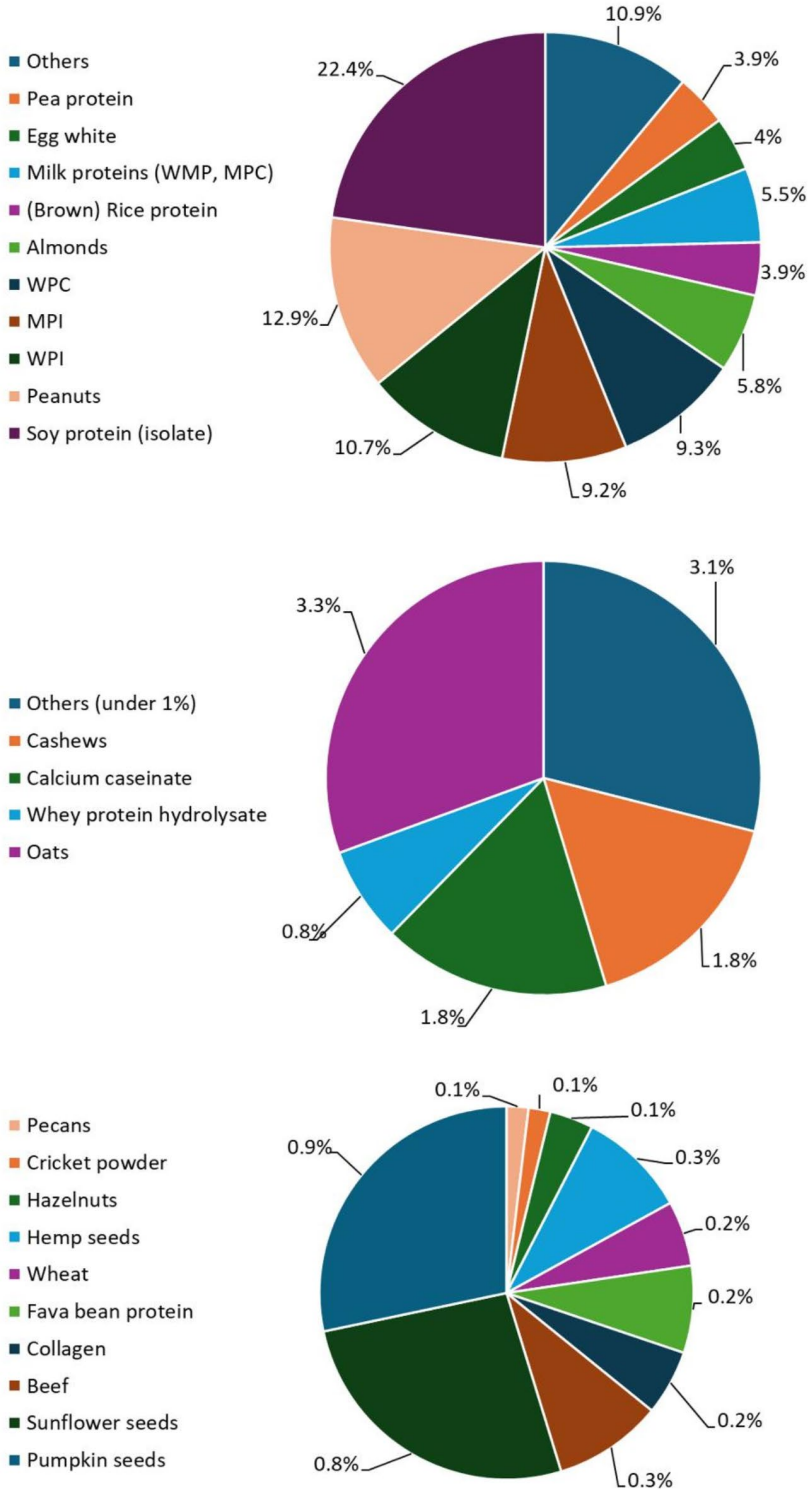
Distribution of protein bars based on primary protein sources. (**A**): Primary protein sources included in the majority of protein bars; (**B**): Primary protein sources present in above 1% of protein bars; (**C**): Primary protein sources present in under 1% of protein bars.

Protein sources of studied 1641 protein bars were identified based on labelling information and protein bars were grouped into 24 categories according to the 24 primary protein sources found (*see list in Methods*). This classification based on primary protein sources is showed on Fig. 1.

The categorization shown on Fig. 1 was based on the frequency of the primary protein sources. From the chosen 24 primary protein sources the first ten gave the 88% of all bars (Fig. 1A), including sources as plant-based proteins; soy protein, rice protein, pea protein, animal-based proteins: milk-based proteins (WPI, MPI, WPC, WMP, MPC) and egg whites, and nuts: peanuts and almonds. The most frequently used protein source was soy protein (isolate) with the relevance of 22%, followed by peanuts (13%) and WPI (whey protein isolate, 11%). The remaining 12% of all primary protein sources were distributed into two larger categories; (i) present in above 1% of all protein bars, such as oats, milk-based proteins (whey protein hydrolysates and calcium caseinate) and cashews (Fig. 1B); and (ii) present in under 1% of all protein bars, some oily seeds (e.g., pumpkin, sunflower, hemp), beef-based proteins (beef and collagen) and special protein sources (e.g., cricket powder) (Fig. 1C).

**Fig. 2:**
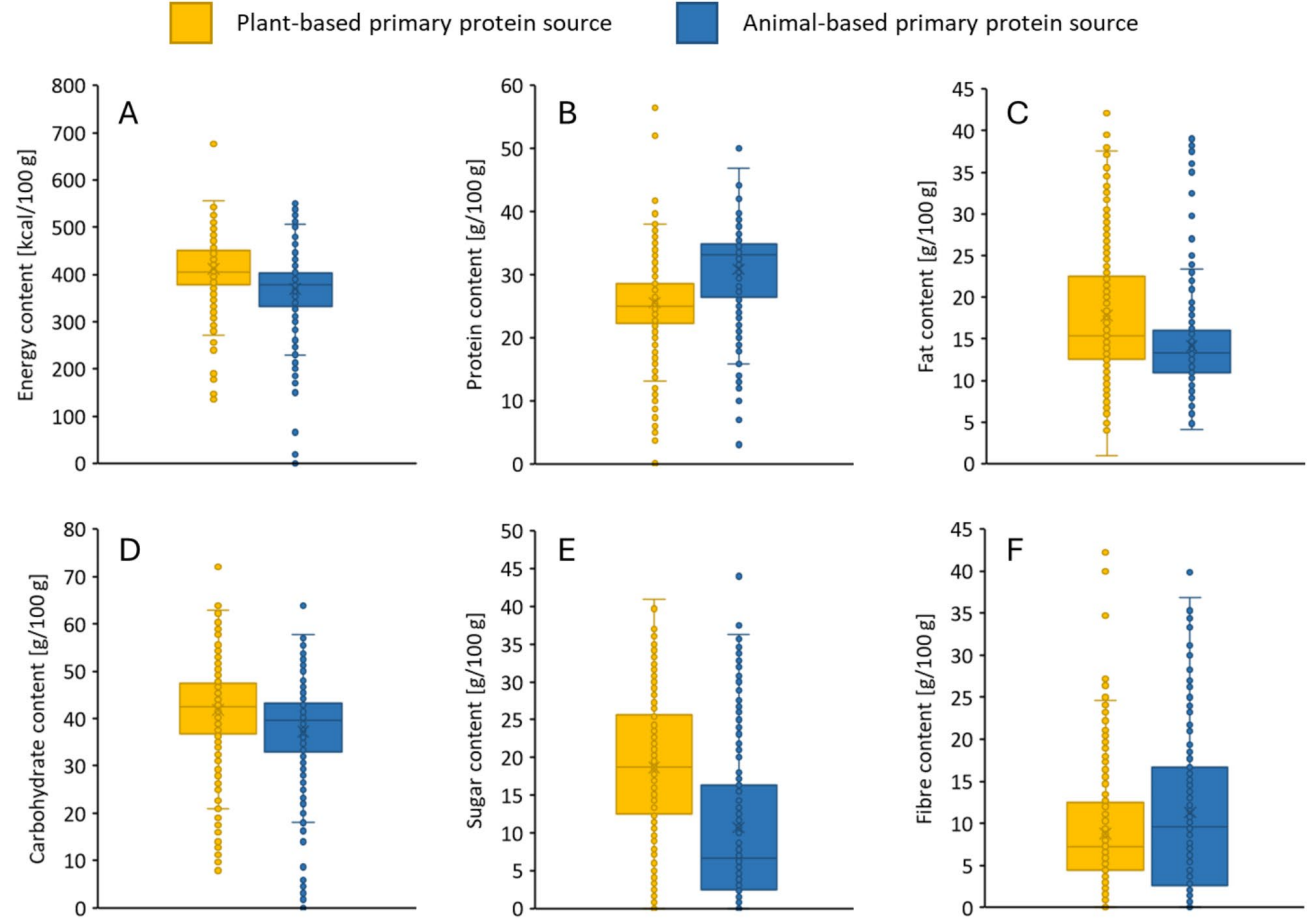
Distribution of energy (**A**; kcal/100 g), protein (**B**; g/100 g), fat (**C**; g/100 g), carbohydrate (**D**; g/100 g), sugar (**E**; g/100 g) and fibre (**F**; g/100 g) content of protein bars sorted according to the quality of primary protein source. Yellow bars show protein bars with plant-based primary protein sources, and blue bars show protein bars with animal-based primary protein sources. Significant difference was stated between animal-, and plant-based bars in all parameters using ANOVA (*p* < 0.001).

Protein bars were also classified based on the type of their primary protein sources, i.e., plant (972 bars) or animal-based (697 bars) (Fig. 2). It can be seen that there is significant difference between the quantity of all evaluated parameters (energy, protein, fat, carbohydrate, sugar, fibre) between animal and plant protein-based bars (Fig. 2; ANOVA; *p* < 0.001). Therefore, there are trends that can be observed e.g., animal-based protein bars have higher protein and fibre content, lower energy, fat, carbohydrate and sugar content.

**Fig. 3:**
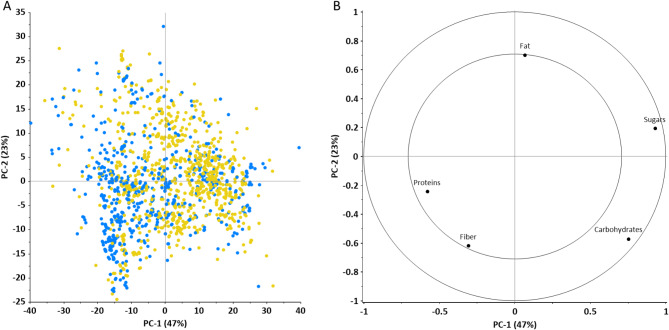
Results of PCA, (**A**): scores plot, (**B**): correlation loadings. Protein bars having plant-based primary protein sources are highlighted with yellow and bars with animal-based protein sources are highlighted with blue.

A principal component analysis (PCA) analysis was also performed in order to reveal the key associations between nutrient profiles and the type of protein source (i.e., animal or plant origin) used in the bars. Figure 3 summarizes the main result of the PCA, the scores plot and the correlation loadings. The samples are also coloured by the protein source (blue – animal-based; yellow – plant-based). The result of PCA showed that the first two principal component (PCs) describe 70% (PC1 47% and PC2 23%) of the explained variance. Based on the results, there was no clear separation among the samples according to the quality of the protein source (animal versus plant) through the PCs.

**Fig. 4:**
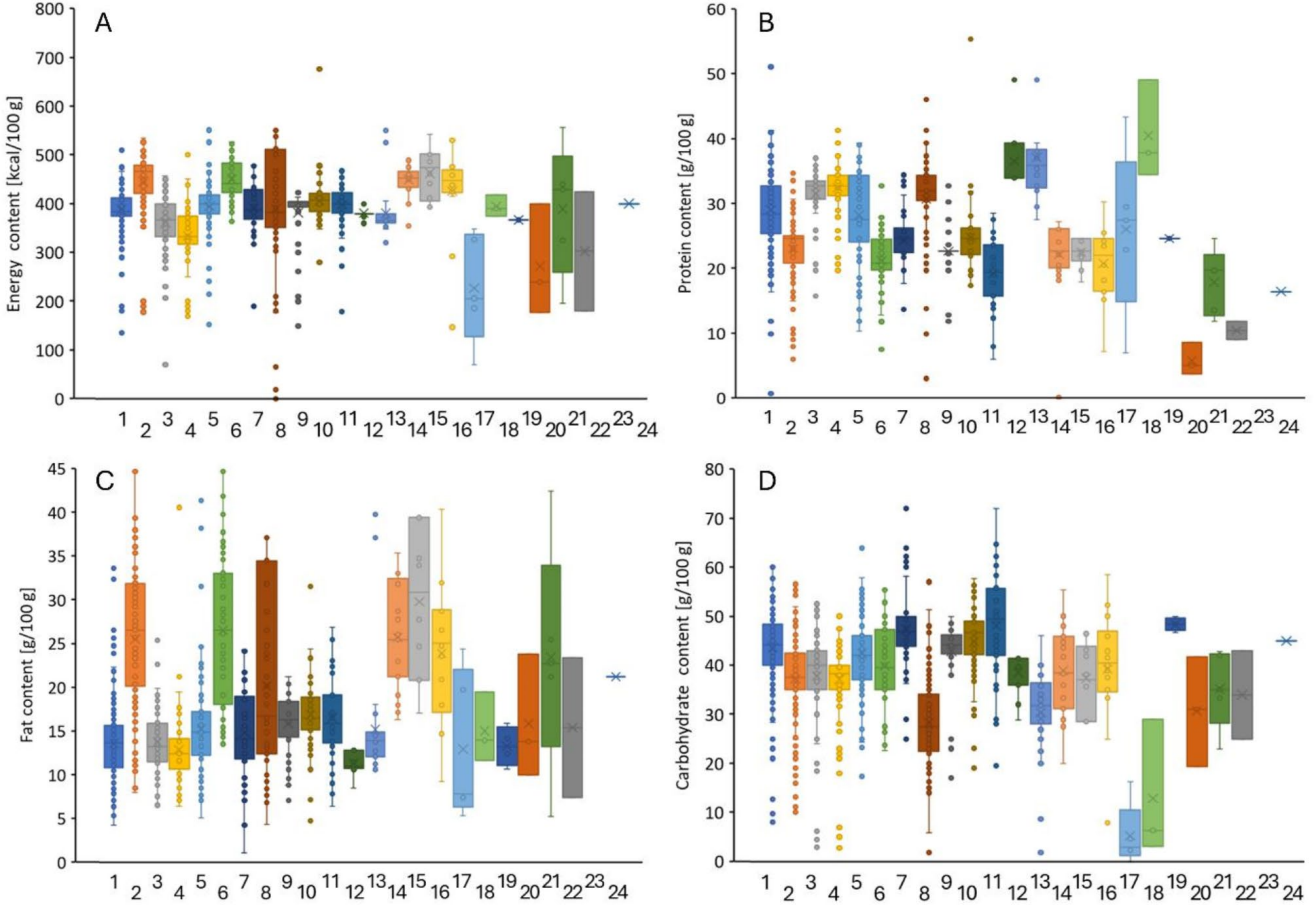
Distribution of energy (**A**; kcal/100 g), protein (**B**; g/100 g), fat (**C**; g/100 g), carbohydrate (**D**; g/100 g) content of protein bars sorted according to the quality of the primary protein source. 1: soy protein (isolate), 2: peanuts, 3: whey protein isolate (WPI), 4: milk protein isolate (MPI), 5: whey protein concentrate (WPC), 6: almonds, 7: (brown) rice protein, 8: milk proteins (WMP: whole milk powder, MPC: milk protein concentrate), 9: egg (white), 10: pea protein, 11: oats, 12: whey protein hydrolysate, 13: calcium caseinate, 14: cashews, 15: pumpkin seeds, 16: sunflower seeds, 17: beef, 18: collagen, 19: fava bean (protein), 20: wheat, 21: hemp seeds, 22: hazelnuts, 23: cricket powder, and 24: pecans.

In addition, based on the protein sources applied, a more detailed classification was also performed regarding energy, protein, fat, carbohydrate contents of protein bars (Fig. 4) and the following associations have been highlighted: (i) protein bars containing nuts (peanuts, almonds, cashews) and seeds (sunflower, pumpkin seeds) have higher fat content; (ii) highest protein content is associated with products containing collagen; (iii) carbohydrate content of protein bars are relatively similar (between 30 and 50 g/100 g) with the exception of bars containing milk proteins (WMP and MPC) and beef originated protein (beef and collagen) which are relatively low, 20–30 g/100 g for milk protein based products and under 30 g/100 g for beef-originated protein bars. These observations are all in accordance with the results of the PCA analysis.

**Fig. 5:**
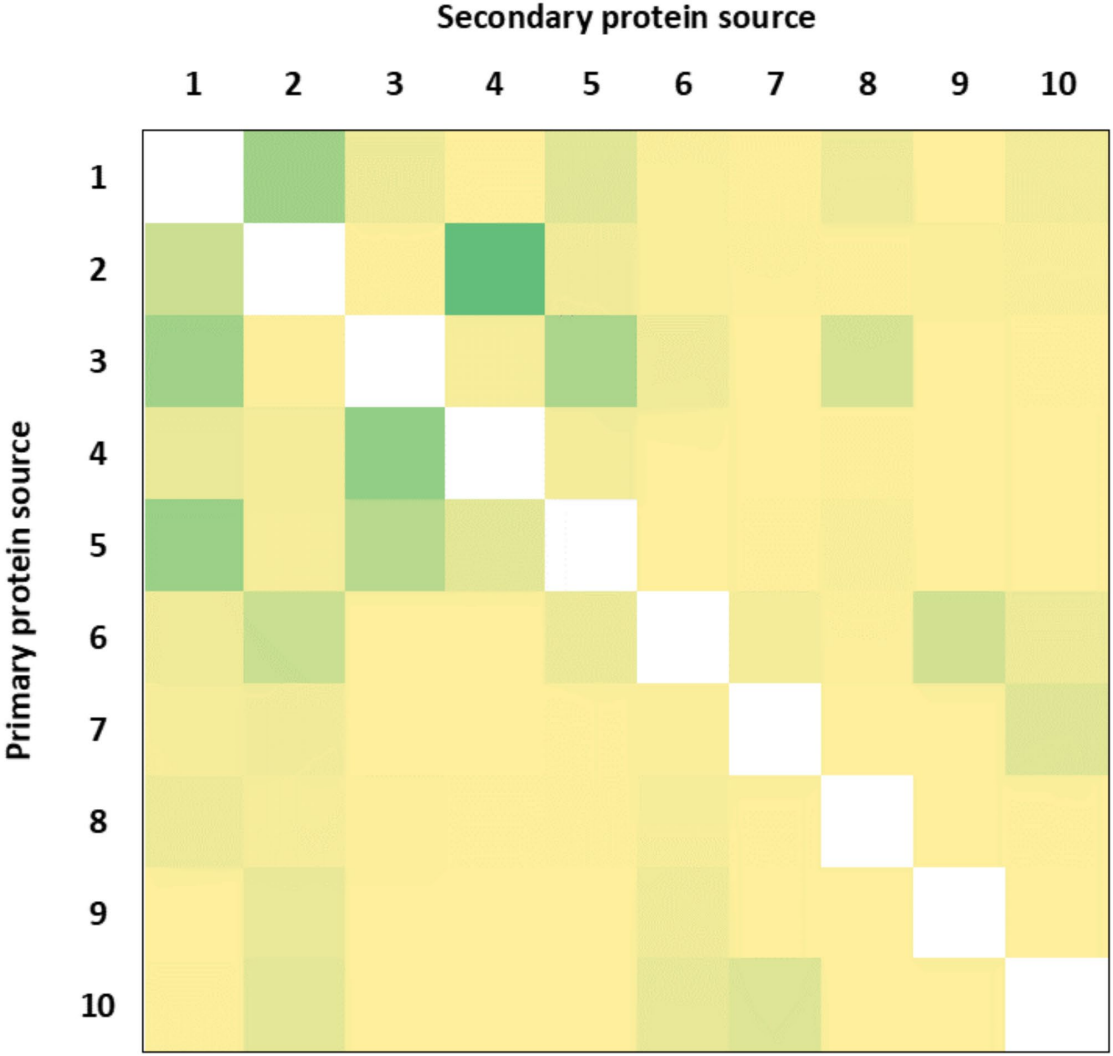
Heatmap of most frequently used protein source combinations. 1: Soy protein (isolate), 2: Peanuts, 3: WPI, 4: MPI, 5: WPC, 6: Almonds, 7: (Brown) Rice protein, 8: Milk proteins (WMP, MPC), 9: Egg white, 10: Pea protein. Green indicates high frequency, yellow shows low frequency.

Those protein bars that contain any of first ten most common protein sources as a primary protein source (1478 bars), were further analysed based on their secondary protein sources (second mentioned protein source in the ingredient list, *for detailed selection criteria see Materials and methods*). The most frequently used protein source combinations are shown in a heatmap in Fig. 5.

Most common pairings were identified and sorted based on quality of protein source (plant-based (211), animal-based (241) and mixed (314) sources). The most common animal protein-based pairings were i) MPI and WPI (86; ii) WPI and WPC (66 + 57); iii) WPI and Milk proteins (WMP and MPC) (32). Amongst the plant-based protein sources the pairings were (i) soy protein and peanuts (75 + 41); (ii) Almonds and peanuts (43); iii), pea protein and (brown) rice protein (27 + 25). In addition, mixed (animal-plant origin) pairings were identified with the most frequent ones being (i) peanuts and MPI (124); (ii) WPC and soy protein (80); (iii) WPI and soy protein (74); (iv) almonds and egg (white) (36).

### Protein nutritional quality of selected bars

Four protein bars products were subjected to simulated digestion using the Infogest protocol^4^ and protein nutritional quality was determined based on the in vitro-DIAAS, an objective indicator according to the method introduced by Sousa et al. with some modifications^22^. In vitro protein digestibility (IVPD%), DIAAR of indispensable amino acids and DIAAS were calculated according to the method described in the expert report published by the FAO (2013)^10^ (*see in Materials and methods*) and results shown in Table 1. Detailed calculations are attached as Supplementary Material S2. Details of the bars are shown in Tables 2 and 3

**Table 1:** In vitro protein digestibility (IVPD%), digestible indispensable amino acid ratios (DIAAR) and digestible indispensable amino acid score (DIAAS) of the four protein bars. Protein sources of the bars are noted.

Parameters	A	B	C	D
Protein sources	PPI and RPI	WPC and MPC	WPI, egg white, calcium caseinate and SPI	milk protein and collagen, peanuts, soy protein
IVPD%	46.7 ± 4.2	85.9 ± 4.3	73.6 ± 9.9	81.2 ± 15.6
DIAAR ^1^				
His	0.40	1.20	0.71	0.70
Ile	0.14	0.75	0.87	1.00
Leu	0.34	0.70	0.72	0.48
Lys	0.52	1.07	0.96	0.89
SAA(Met + Cys)	0.13	0.43	0.52	0.85
AAA (Tyr + Phe)	0.33	0.49	0.32	0.16
Thr	0.44	0.66	0.27	0.04
Trp	0.32	0.28	0.16	0.16
Val	0.36	0.90	0.83	0.92
DIAAS% (LAA) ^1^	13 (SAA)	28 (Trp)	16 (Trp)	4 (Thr)
DIAAR ^2^				
His	0.42	1.26	0.75	0.74
Ile	0.24	1.29	1.49	1.72
Leu	0.49	1.02	1.04	0.70
Lys	0.63	1.29	1.17	1.08
SAA(Met + Cys)	0.16	0.52	0.64	1.04
AAA (Tyr + Phe)	0.60	0.88	0.57	0.29
Thr	0.62	0.93	0.38	0.06
Trp	0.65	0.56	0.33	0.32
Val	0.46	1.15	1.06	1.18
DIAAS% (LAA) ^2^	16 (SAA)	52 (SAA)	33 (Trp)	6 (Thr)
DIAAR ^3^				
His	0.52	1.58	0.94	0.92
Ile	0.25	1.37	1.59	1.84
Leu	0.53	1.11	1.13	0.76
Lys	0.75	1.53	1.38	1.28
SAA(Met + Cys)	0.19	0.61	0.75	1.22
AAA (Tyr + Phe)	0.75	1.12	0.72	0.36
Thr	0.77	1.15	0.47	0.07
Trp	0.84	0.72	0.42	0.41
Val	0.50	1.24	1.14	1.27
DIAAS% (LAA) ^3^	19 (SAA)	61 (SAA)	42 (Trp)	7 (Thr)

DIAAR calculations were based on indispensable amino acid 1: reference pattern for age group 1, infant (0–6 month); 2: reference pattern for age group 2, child (6–36 month); 3: reference pattern for age group 3, older child, adolescent and adult set in the report published by FAO 2013^10^.

LAA: limiting amino acid; PPI: pea protein isolate; RPI: rice protein isolate; WPC: whey protein concentrate; MPC: milk protein concentrate; WPI: whey protein isolate; SPI: soy protein isolate.

**Table 2:** Ingredient list of protein bars. Protein sources are highlighted with bold.

Bars	Ingredients
Bar A	Protein blend (pea protein isolate, rice protein concentrate), fructo-oligosaccharide, isomalto-oligosaccharide, cocoa coating mass with sweetener (20%) [sweetener (maltitol), vegetable fat (palm, palm kernel, shea), fat-free cocoa powder (13%), emulsifiers (lecithins)], emulsifier (soy lecithin), rapeseed oil, pea protein crunch (8%)(pea protein, tapioca starch), cocoa powder, chocolate chips (2.3%) [cocoa mass, sweetener (maltitol), cocoa butter, emulsifier (soy lecithin), natural vanilla flavouring], humectant (glycerol), flavourings, antioxidant (rosemary extract), sweetener (steviol glycosides).
Bar B	Moisturizers (maltitol, glycerol), coating [sweetener (maltitol), fully hydrogenated vegetable fats (coconut, rapeseed), low-fat cocoa powder, emulsifier (lecithins)], whey protein concentrate, milk protein concentrate, fructo-oligosaccharides, hydrolysed collagen, refined coconut oil, whey protein isolate [whey protein isolate, emulsifier: lecithins (soy)], drinking water, almond flour, emulsifier: lecithins (soy), solidifying agent (carrageenan), flavourings, non-fat cocoa powder, table salt, preservative (potassium sorbate), stabiliser (potassium chloride), antioxidant (alpha-tocopherol), sweetener (sucralose).
Bar C	Protein blend (whey protein isolate (milk), egg protein, hydrolysed protein (milk), calcium caseinate (milk), soy protein isolate), humectant (glycerol) (contains soy), maltitol), palm fat, fructo-oligosaccharides, reduced fat cocoa powder, extruded soy, Chocolate shavings (maltitol sweetener, cocoa mass, soy lecithin emulsifier, reduced fat cocoa powder, natural flavouring), water, soy lecithin emulsifier, flavourings, gelling agents (carrageenan, potassium chloride), preservatives (potassium sorbate), cinnamon, DL-alkatophorol acetate, sweetener (sucralose).
Bar D	MALTIT-MILK CHOCOLATE 23% (sweetener: maltitol; cocoa butter, whole milk powder, cocoa mass, emulsifier: soy lecithin; flavourings), milk protein, collagen hydrolysate, bulking agent: polydextrose; peanuts 10%, humectant: glycerol; soy protein, soybean oil, sweetener: xylitol, sucralose; flavourings, table salt, sunflower oil, emulsifier: soy lecithin.

**Table 3:** Nutritional information of the four protein bars according to the packaging. Protein sources of the bars are noted.

Nutrition Information Per Serving (100 g)				
Code	Bar A	Bar B	Bar C	Bar D
Protein sources	PPI and RPI	WPC and MPC	WPI, egg white, calcium caseinate and SPI	milk protein and collagen, with peanuts, soy protein
Energy (kcal)	402	370	380	373
Fat (g)	20.4	17	15	17
Of which saturated(g)	8.2	13	8.5	6.8
Carbohydrates (g)	22.3	31	22	26
Of which sugars (g)	4.9	2.6	1.9	3
Of which polyols (g)	12.8	-	17	21
Dietary fibre (g)	21.4	7.5	12	10
Protein (g)	28.6	30	40	34
Salt (g)	0.57	0.57	0.8	1.1
Protein ratio (E%)	29.5	30.2	41.8	34.6
Category of bar	“high in protein”			

PPI: pea protein isolate; RPI: rice protein isolate; WPC: whey protein concentrate; MPC: milk protein concentrate; WPI: whey protein isolate; SPI: soy protein isolate.

DIAAR calculations were based on indispensable amino acid 1: reference pattern for age group 1, infant (0–6 month); 2: reference pattern for age group 2, child (6–36 month); 3: reference pattern for age group 3, older child, adolescent and adult set in the report published by FAO 2013^10^. LAA: limiting amino acid. PPI: pea protein isolate; RPI: rice protein isolate; WPC: whey protein concentrate; WPI: whey protein isolate; MPC: milk protein concentrate; SPI: soy protein isolate.

Highest IVPD% and DIAAS was measured for the protein bar only containing animal-based protein sources (bar B: 85.9 ± 4.3%, age group 1: 28 (Trp); age group 2: 52 (SAA); age group 3: 61 (SAA). Protein content of this bar is composed of WPC (whey protein concentrate) and MPC (milk protein (casein) concentrate), the two types of proteins found in milk.

From the results in vitro PDCAAS of products were also calculated (Eq. 3). The values for the age group 3 (older child, adolescent, adult) are as follows; Bar A: 44 (Trp), Bar B: 62 (Trp), Bar C: 33 (Trp), Bar D: 34 (Trp). It was concluded that all four products have the limiting AA of Trp and with the correction with IVPD% value this results in low PDCAAS value for all four protein bars.

## Discussion

OpenFoodFacts.org is a product of citizen science, in particular a database of packaged foods containing comprehensive information on ingredients, nutrient content, allergens and more complex indexes like NutriScore (based on nutritional composition), NOVA (based on level of processing) and Eco score (based on carbon footprint). The database contains reliable information (i.e., information derived from nutrition labelling of products and validated by photos) on food products that are uploaded by conscious consumers. Using the collected data, our first question addressed the eligibility of protein claims of the bars marketed as “protein bars”. It has been proposed that the sometimes misleading advertisement using phrases like “high protein” and “rich in protein” depicted on packaging of protein bars could shift consumer perception into falsely make a “healthier” food choice^11^. Protein content of studied bars was calculated according to the 1924/2006/EU regulation on nutritional labelling of food products in the percentage of the energy (E%)^23^. Categories were set *“not protein source”*: <12E%; *“protein source”*: >12E% and < 20E%; *“high in protein”*: >20E%. According to our results, it can be stated that the majority (81%) of the protein bars in the database have sufficiently high protein content and appropriate nutrition profile to satisfy the claim “*high in protein*” according to EU regulations.

The protein content of protein bars mostly covered by the addition of isolated protein sources i.e., protein powders such as whey or milk protein isolates or soy protein isolate. The detailed analysis of primary protein sources (Fig. 1) revealed that the most used plant-based protein source is soy protein (isolate) and the most used animal-based protein source is whey protein isolate. This is in accordance with the sparse literature data on protein bars stating that soy and whey proteins are used as the most commonly used ones for protein bars due to their good techno functional properties^5,14^. However due to the sear nature of protein powders, recipes should include several additives to enhance flavour, texture and mouthfeel and to create a palatable product with low water content for longer storage^19^. These include fibres (fructo-oligosaccharides), emulsifiers (lecithin), humectants (maltitols, glycerine), gelling agents (carrageen, potassium chloride) or volume enhancers (polydextrose)^16^. These additional components and their ratio largely depend on the protein source(s) used as a base for the protein bars and not only could modify the rheological properties of protein bars but the nutritional composition and nutritional quality as well. Therefore, two additional research questions were proposed. (i) Is there a link between the source of the protein and the nutritional composition (energy, protein, carbohydrate, sugar, fat and fibre contents) of the protein bars? (ii) How do these additional components modify the perceived protein nutritional quality of the most frequently used protein source combinations?

Based on our results given in Figs. 2 and 3, the following trends are become visible. Animal-based protein bars have higher protein and fibre content, lower energy, fat, carbohydrate and sugar content. The differences in composition of products containing proteins of animal origin or plant origin are also indicated to some extent in the performed PCA analysis. Since the protein content of the products is almost identical, the difference between them is determined by the other ingredients (carbohydrate, sugar, fat and fibre content). The separation of the samples (Fig. 3A) was mostly affected by the carbohydrate and sugar content that was shown by the correlation loadings (Fig. 3B). Samples with higher carbohydrate and sugar content were located on the positive range of the PC1, while samples with higher protein and fibre content on the negative range. However, the protein and fibre content had smaller effect on the location of the samples through both PCs. The fat content had also considerable effect on the separation of the samples based on the PC2. Samples with higher fat content were located on the positive side. The plant-based protein bars mostly can be found on the positive side of the PC1 that means these have higher carbohydrate and sugar content. A more detailed analysis on the interrelation of the protein source used and the composition (see Fig. 4) revealed that highest protein content is associated with products containing added collagen. Further observations regarding the nutritional relevance of collagen, which is preferred for the purpose of introducing the protein content into protein bars, will be discussed in more detail later.

Almost half (47%) of the studied protein bars contain multiple sources of proteins. Protein bars were analysed by their primary and secondary protein sources in order to reveal the most often used protein ingredients in the bars. We could identify products containing protein ingredients only of plant origin, only of animal origin or mixed sources. Overall, the most popular protein source combination was peanut and MPI with 124 mentions however it only concluded 8% of the analysed bars. Most common pairings for further analysis were chosen: (i) milk and whey proteins; (ii) pea and rice proteins; (iii) whey and soy proteins; (iv) milk protein and peanuts.

In addition to database evaluations, the four selected protein bars (A, B, C, D) were subjected to further analysis of the protein nutritional quality. Protein nutritional quality was characterised and quantified using the in vitro DIAAS and in vitro PDCAAS values. Results of the evaluation of protein source pairings among analysed protein bars served as a base for the selection of the four protein bar products. According to the in vitro digestion experiments, it was shown that the protein bars containing proteins of animal origin have a greater digestibility (IVPD% between 73.6 and 85.9) compared to bar A containing only plant (pea and rice) proteins (46.7). This observation is in agreement with the generally accepted view that animal proteins have higher nutritional quality compared to plant proteins^13,20^. In particular, the data shared by Mathai et al. (2021)^18^ the in vivo standardized ileal digestibility (SID) of WPC (whey protein concentrate) and MPC (milk protein (casein) concentrate), are 98% and 92%, respectively. This high digestibility of milk proteins also seems to be true for these protein sources even in a highly complex matrix as the protein bars. However, overall digestibility of these proteins was decreased to some extent by their inclusion into the protein bars.

The PDCAAS value is based on the amino acid composition of the product and only includes this average in vitro digestibility into calculated protein nutritional quality assessment. Therefore, the relatively low digestibility values due to the matrix effect would be reflected in the PDCAAS. PDCAAS results show that Trp is the limiting AA in all cases and all values are below 0.75.

Nevertheless, the DIAAS values of the bars show much pronounced decrease in all age groups compared to the DIAAS reported (133 (His) and 141 (SAA), respectively (for age group 2). Our results show that after the in vitro digestion simulation, the release of individual amino acids from the matrix of the protein bar resulted in a DIAAS of only 52 (SAA) for the mixture of the two animal-based protein sources (age group 2). Therefore, it seems that the slight decrease in IVPD% resulted in a much prominent decrease in DIAAS. This means that the liberation and release of some amino acids (in particular SAAs and Trp in the case of milk proteins) during digestion is significantly lower than the IVPD% that characterizes the gross average digestibility of the whole protein. In other words, in the case of milk proteins, the SAAs and Trp that are present either in their free form or remained in peptides and are, for example, associated with matrix constituents, are not taken into account during the DIAAS determination.

These results highlight the advantages of amino acid-based evaluation of protein digestibility especially if exploring the causes of poorer digestibility can also be interesting. Since PDCAAS does not account for the different digestibility patterns of individual amino acids i.e., lower release of SAA from the matrix of the protein bar A. Therefore, the complexity of the information obtained from DIAAS values is higher than from the PDCAAS. Nevertheless the above discussed food matrix effect on protein digestibility is also indicated to some extent by the relatively low PDCAAS values for the protein bar A (48 (Trp) for age group 2) compared to the value for the individual protein sources WPC (107 (His) and MPC (121 (SAA)^18^.

This trend also could be observed for the other selected protein bars. Although digestibility of plant-based protein sources are generally accepted to be lower, Baley and colleagues^1^ found that both rice and pea protein concentrates have relatively high SID (79% and 93%). However, despite the adequate digestibility, the DIAAS of both protein sources measured on the lower side (30 (Lys) and 60 (SAA) for age group 2). Since these values are still higher than the DIAAS for bar A (Table 1) it is hypothesized that although pea and rice proteins could complement each other’s amino acid profile due to the complex matrix of the protein bar the digestibility of amino acids could be reduced. Moreover, if only PDCAAS were to be observed for protein bar B this information would have been overlooked since in vitro PDCAAS is closer to the individual protein sources’ values than as observed for the bar itself (34 (Trp) for age group 2).

Bar C and D both composed of a mixture of several protein sources including both animal and plant-based proteins. This is reflected in their digestibility (both higher than 70%) however DIAAS was even lower than expected. The most extreme values were measured for the protein bar D. Although digestibility is relatively high (81%) DIAAS is below 10% in all of the age groups with LAA being threonine. This bar contains milk protein, collagen, peanuts, and soy protein from which milk protein and collagen being the main sources of proteins. It was shown that protein bars containing collagen are on the high end in terms of protein content (35–50 g/100 g). However collagen mostly composed of non-essential amino acids i.e., glycine, glutamine, proline, hydroxy proline with a lower level of indispensable amino acids (under 2.5 g/100 g of protein^12^), . This fact explains the measured very moderate DIAAS value for bar D, since practically in the DIAAS calculation, the amount of essential amino acids present in a unit amount of total protein determines the DIAAR values and ultimately the DIAAS value. This results in the scenario when the amino acid profile of collagen, which is poor in essential amino acids, does not contribute to the amount of amino acids that would result in an increase in the DIAAS value, although amounts of added collagen should be taken into account in terms of the total amount of protein. Therefore, in the case of protein bars containing collagen the protein content per se might not be a relevant attribute to assess protein nutritional quality, due to their very moderate DIAAS. It is noted that although PDCAAS values are relatively low for these bars, however the specific effect of the addition of collagen – being poor in indispensable amino acids – is not manifested in the PDCAAS results.

It should be noted however that regarding the protein claims regulated by the EU^23^ all four protein bars are eligible for “*high in protein*” claim. However according to the FAO protein quality guidelines^10^ it is recommended that no nutrition claim should be allowed to be made for source/high protein for proteins with DIAAS (and PDCAAS) less than a certain cut-off (e.g. 75). Eventually it is also noted that it may seem that animal protein-based protein bars have better nutritional composition e.g., higher protein, lower carbohydrate and fat content, it could not be forgotten that animal proteins require larger economical footprint to be produced than plant proteins. In the work of Coluccia et al., the introduction of an indicator called Carbon Footprint Protein Ratio (CFPRO) was made by the integration of protein quality, quantity and their average carbon footprint (in kg CO_2_-eq)^7^. This indicator is based on the DIAAS, the serving size and the protein ratio of the food and helps to evaluate foods in term of both nutrition and sustainability^2^. In this work, they have compared the environmental impact corrected protein nutritional score of cow’s milk and soy drink, and showed that although soy drink has lower DIAAS thus poorer protein nutritional quality it’s also lower carbon footprint results in better CFPRO score and lower overall impact on the environment than cow’s milk.

## Conclusion

Evaluation of commercially available protein bars were done focusing on protein content, protein source(s) and nutritional content using consumer-generated online data. Overall, it was revealed that protein claims regulated by the EU could be applied on most of the protein bars included in the analysis. Moreover, protein content is greatly affected by the additional ingredients such as carbohydrates, fats and fibres as well as the quality of protein source i.e., plant or animal-based protein source was used as primary protein source. In order to gain more detailed and scientifically current opinion on protein nutritional quality of the protein bars the FAO recommended objective indicator, the DIAAS was measured for four selected protein bars with diverse quality of protein sources. These bars were included based on the quality of the combination of primary and secondary protein sources. It was shown that although protein claims regulated by the EU could be applied on all four protein bars according to their protein content still all bars have lower DIAAS than 75% therefore no protein quality claims should be made according to the FAO protein quality guidelines. The decrease in DIAAS was also prevalent even when the in vitro protein digestibility has not changed drastically compared to the individual protein source’s digestibility. Although the inclusion of multiple protein sources could have the complementary effect on amino acid composition of the protein bars, the additional ingredients necessary to create a consumer-accepted and nutritionally more complex product may hinder digestibility of protein bars on the amino acid level. Therefore, we suggest the consumption of protein bars as a part of a complete diet. We think that these results raise the question of the nature of the true protein nutritional quality of high protein products and generate the need for more data to reveal the relationship between protein sources, the effect of additional ingredients and protein nutritional quality of protein-associated food products.

## Methods

### Materials

Materials for digestion protocol, pepsin from porcine gastric mucosa (> 2500 units/mg protein), pancreatin from porcine pancreas (8xUSP), porcine bile extract were purchased from Merck/Sigma-Aldrich (Merck & Co., Philadelphia, PA, USA). Methanol (CHROMASOLV™, gradient grade, for HPLC, ≥ 99.9%) was produced by Riedel-de Haën (Honeywell International Inc., Charlotte, North Carolina, US).

### Data collection and curation

Data was retrieved in January 2023 from O*penFoodFact.org* using the keyword “protein bars”. The search term found 4638 products (*uncurated data can be found as Supplementary Material S1*), which were narrowed down based (i) on the availability of ingredient list in English language; and by (ii) availability of quantitative information studied major nutrients. The initial database containing 4638 products was narrowed down to 1669 products, which have a complete ingredient list available in English. Further 26 records were excluded due to lack of compositional data on fat and carbohydrates (including sugars) and proteins. Data of the remaining 1641 products was used as a generic database for all analysis except for the ones relating to fibre content. For that purpose, a subset of the generic database containing 1570 products was used, since 73 further products of the generic database should be excluded due to the lack of quantitative data on fibre content.

The categorization of protein bars based on protein content was done according to the 1924/2006/EU regulation^23^ on nutritional labelling of food products. Categories were set *“not protein source”*: <12E%; *“protein source”*: >12E% and < 20E%; *“high in protein”*: >20E%.

When classifying protein sources, protein ingredients were chosen on the assumption that their addition into the recipe was in order to enhance protein content of the bars, despite their relative (high or low) protein content, e.g., fava bean or fava bean protein. Fava bean is usually a source of protein however protein content of the source is not declared in the ingredient list. Therefore, every mention of fava bean was noted as protein source, i.e., four bars contained fava bean or fava bean protein as primary protein source. The sum of 24 protein ingredients were chosen, namely; soy protein (isolate), peanuts, whey protein isolate (WPI), milk protein isolate (MPI), whey protein concentrate (WPC), almonds, (brown) rice protein, milk proteins (WMP: whole milk powder, MPC: milk protein concentrate), egg (white), pea protein, oats, whey protein hydrolysate, calcium caseinate, cashews, pumpkin seeds, sunflower seeds, beef, collagen, fava bean (protein), wheat, hemp seeds, hazelnuts, cricket powder, and pecans.

Primary protein source: first mentioned protein ingredient was noted as primary protein source of each bar. Secondary protein source: protein ingredient mentioned secondly was noted as secondary protein source, however if a protein source ingredient was mentioned as part of another ingredient (i.e., coatings or decorations usually contain whey or milk protein) these sources were not counted as protein source. Secondary protein sources were assessed for the ten most common primary protein sources to determine the most common complementation amongst protein ingredients. Most common pairings were chosen based on number of bars in the category.

### Samples

Four, commercially available protein bars were selected based on the quality of their protein sources to assess the relationship between protein source and protein nutritional quality. Selected bars were purchased in Budapest, Hungary at several small shops. Bars were coded as follows: (A) plant only (pea and rice); (B) animal only (milk proteins); (C) mix of animal (milk and egg) and plant (soy); (D) mix of animal (milk and collagen) and plant (soy) Ingredient list of protein bars and nutritional information of bars were noted from the packaging could be found in Tables 2 and 3, respectively. Before digestion simulation whole bars were homogenized using an electric coffee grinder (Bosch TSM6A013B type grinder, Robert Bosch GmbH, Gerlingen, Germany), grinding was repeated for three times for approximately 5 s, in order to simulate oral mastication. Samples were stored at -80 °C and thawed before digestion simulation.

In addition, protein content of bars was determined using the Kjeldahl method. First, protein bars were defatted using cold organic solvent extraction. Homogenized protein bars (20 g) were measured into Erlenmeyer flasks and 80 ml hexane was added. Extraction took place for 4 h at room temperature with mixing at every 20 min then samples were sieved and dried (45 min at 55 °C). Into the Kjeldahl digestion tubes 0.25 g of defatted and homogenized samples were measured, 1 piece of Kjeldahl tablet (Molar Chemicals Kft., Halásztelek, Hungary) and 20 mL of 98% sulfuric acid (VWR Chemicals, Radnor, Pennsylvania, USA) were added, mixed and heated for 3 h in a Gerhardt Kjeldatherm equipment (Gerhardt GmbH & Co. Königswinter, Germany). After acidic digestion vapour distillation was carried out in a Gerhadt Vapodest 45s (Gerhardt GmbH & Co. Königswinter, Germany) and titration was conducted using 0.05 M sulphuric acid solution. Protein content was calculated based on the nitrogen content using the conventional conversion factor 6.25.

Amino acid composition of the protein bars were measured after microwave-assisted digestion similar to the digests. From the defatted protein bars 10 mg was measured and dissolved in 0.5 mL 6 M HCl (with 1% phenol) and 0.25 mL was transferred into the rotor of the digestion vessel. After microwave digestion, samples were transferred to glass reaction tubes, taken up with 1 M Na-borate buffer, pH was set to 8.5 and volume was adjusted to 5 mL and filtered using 22 μm HPLC filter prior to derivatization. Derivatization, separation and quantification methods are described later (see section on *Protein nutritional quality*). Amino acid composition of products is showed in Supplementary Materials S2.

###  In vitro digestion simulation

Protein nutritional quality was determined based on amino acid content of ileal digesta after simulated digestion using the Infogest method^4^ based on the modifications made by Sousa and colleagues^22^. Sample size was normalized based on protein content of the bars, set to 40 mg protein per digestion tube and diluted with water to 1 g. To the 1 g of diluted sample 0.8 mL simulated salivary fluid (pH 7, 37 °C), 0.005 mL 0.3 M CaCl_2_ and 0.195 mL water were added. Right after, 1.28 mL simulated gastric fluid (pH 3, 37 °C), 0.001 mL 0.3 M CaCl_2_, 0.32 mL pepsin stock solution (2000 U/mL of digesta) were added. The pH of gastric phase was set by the addition of 6 M HCl at different volumes. Water was added to reach 4 mL end-volume of gastric phase and samples were incubated for 2 h at 37 °C in an overhead shaker (Heidolph Reax 2; Heidolph Instruments, Schwabach, Germany). Before the small intestinal phase, 1.7 mL simulated intestinal fluid, 0.008 mL 0.3 M CaCl_2_, 0.5 mL bile solution (160 mM) and 1.0 mL pancreatic enzyme stock solution (100 U/mL trypsin activity in digesta) was added. Addition of 1 M NaOH was not necessary to set the pH of samples therefore 0.792 mL of water was added to each sample and digestion continued for an additional 2 h. Blank digestion – for correction with enzyme originated background amino acid content – was carried out using a protein-free cookie as a sample. The protein-free cookie was made according to Moughan and colleagues^21^, stored at -80 °C, thawed before the experiments and 1 g was measured for in vitro digestion simulation.

### Protein nutritional quality

Determination of protein nutritional quality was based on the method established by the FAO Expert Consultation^10^ and the method of Sousa et al.^22^. However, in our calculation DIAAR and DIAAS were calculated from the amino acid profile of the digests. We propose this facilitated calculation method based on a few methodological differences. The calculation protocol proposed by the FAO is based on the methodology of the in vivo experiments, i.e., the amino acid profile of the remaining undigested and not absorbed fraction could be measured. Moreover, the digested and absorbed fraction’s composition only could be acquired if the product’s amino acid composition is known. Nonetheless, in this case, the determination of the product’s amino acid composition is necessary to calculate the amino acid composition of the bioavailable protein fraction. However, after in vitro digestion simulation the amino acid composition of the bioaccessible fraction could be directly assessed, i.e., all amino acids present at the place of absorption. Therefore, amino acid-based protein nutritional quality could be directly determined form the amino acid composition of the selectively isolated digesta (*Supplementary Material S2*).

Sample preparation for determination of in vitro DIAAS was according to Sousa et al.^22^. After the end of the small intestinal phase 32 mL of methanol was added to each tube in order to precipitate undigested proteins. This also acted as a deactivation step for the enzyme activity. Samples were placed at -20 °C for 1 h afterwards pellets were separated using centrifugation (4000*g*, 20 min, 4 °C; MWP – 260R type centrifuge, MWP Med. Instruments, Warsaw, Poland), supernatant was collected in a clean centrifuge tube and stored at -20 °C for further analysis.

Amino acid composition was determined after complete hydrolysis of the supernatant containing digested protein fraction using microwave-assisted digestion (Milestone Ethos Up type high performance microwave digestion system, Milestone™ Srl, Sorisole, Italy) with two methods for (i) general amino acid composition excluding tryptophan (heating time 10 min to 160 °C with 20 min incubation time); (ii) tryptophan-specific method (heating time 10 min to 160 °C with no incubation time). A 1.0 mL portion of the homogenized supernatant was transferred into 2-mL microtubes and solvent was evaporated in a vacuum centrifuge (ScanVac vacuum centrifuge with CoolSafe cooling system, Scientific Laboratory Supplies Limited, Nottingham, United Kingdom). Before microwave digestion the residue was resolved in 0.5 mL 6 M HCl (with 1% phenol) and 0.25 mL was transferred into the rotor of the digestion vessel. After microwave digestion, samples were transferred to glass reaction tubes, taken up with 1 M Na-borate buffer, pH was set to 8.5 and volume was adjusted to 2.5 mL and filtered using 22 μm HPLC filter prior to derivatization. Derivatization process using AQC-reagent was according to the method developed by Waters (Waters Corp., Milford, Massachusetts, USA). Sample (0.010 mL) was added to 0.070 mL of 1 M Na-borate buffer and 0.020 mL of Waters AccQTag reagent (AQC), incubated at a temperature of 55 °C for 10 min and filtered with a 22 μm HPLC filter. Chromatographic analysis was carried out using a Waters Acquity UPLC H-Class fitted with an AccQ UPLC BEH C18 column (2.1 × 100 mm, 1.7 μm). The column was kept at a temperature of 43 °C. Each sample was injected with a volume of 10 µL and a flow rate of 0.7 mL/min. A PDA detector – set to 260 nm – was used to detect individual amino acids after separation. An assessment of both the quality and quantity was performed by using amino acid standards.

Equation 1 was used to calculated in vitro protein digestibility (IVPD%). Equation 2 was used for calculation of DIAAR (digestible indispensable amino acid ratio). Reference amino acid pattern was cited from the Report on FAO Expert consultation (2013)^10^ shown in Table 4. The lowest value of DIAAR’s was set as the DIAAS (digested indispensable amino acid score). DIAAS was given as ‘DIAAS% (limiting amino acid)’.

In addition, PDCAAS was calculated according to Eq. (3). based on the amino acid profiles of products and the IVPD% (calculated according to Eq. 1). It is also noted that PDCAAS above 100% should be truncated if necessary due to methodological considerations. Reference amino acid pattern was cited from the Report on FAO Expert consultation (2013)^10^ shown in Table 4 as seen in the work of Mathai et al.^18^. PDCAAS was given as ‘PDCAAS% (limiting amino acid in product)’.

 Calculation of in vitro protein digestibility (IVPD%).

1 $$\:IVPD\%=\:\left(\frac{sum\:of\:digested\:amino\:acids\:\left[\frac{g}{100\:g}product\right]}{protein\:content\:\left[\frac{g}{100\:g}\:product\right]}\right)*100$$

Calculation method for DIAAR.

2 $$\:DIAAR=\left(\frac{amino\:acid\:content\:of\:digest\:\left[\frac{mg}{g}protein\right]}{reference\:amino\:acid\:content\:\left[\frac{mg}{g}protein\right]}\right)$$

Calculation methods for PDCAAS.

3 $$\:PDCAAS=\:min\left(\frac{amino\:acid\:content\:of\:product\:\left[\frac{mg}{g}protein\right]}{reference\:amino\:acid\:content\:\left[\frac{mg}{g}protein\right]}\right)*IVPD\%$$

**Table 4:** Reference Amin acid pattern from FAO report 2013^10^.

	Age groups		
AA	Infant (0–6 month)	Child (6–36 month)	older child, adolescent, adult
Histidine (His)	21	20	16
Isoleucine (Ile)	55	32	30
Leucine (Leu)	96	66	61
Lysine (Lys)	69	57	48
SAA (methionine + cysteine)	33	27	23
AAA (tyrosine + phenylalanine)	94	52	41
Threonine (Thr)	44	31	25
Tryptophan (Trp)	17	8.5	6.6
Valine (Val)	55	43	40

### Statistical analysis

Data analysis was carried out in Microsoft Excel (Version 2405 64-bit; Microsoft Corporation, Albuquerque, NM, USA). One-way ANOVA was done to assess difference between nutritional composition (protein, fat, carbohydrate, sugar, and fibre) of animal and plant-based protein bars using IBM SPSS Statistics software (Version 29.0.1.0 (171), International Business Machines Corporation, Armonk, NY, USA). Principal component analysis (PCA) was performed among the protein bars for pattern recognition based on the composition. The validation of the model was done with random eleven-fold cross-validation by using The Unscrambler X 10.4. software (CAMO, Oslo, Norway). NIPALS algorithm was used due to some missing values in the nutritional data of the protein bars.

## Electronic supplementary material

Below is the link to the electronic supplementary material.

Supplementary Material 1

Supplementary Material 2

## Data Availability

Data is publicly available at openfoodfacts.org.

## References

[1] Bailey, H. M., Fanelli, N. S. & Stein, H. H. Effect of heat treatment on protein quality of rapeseed protein isolate compared with non-heated rapeseed isolate, soy and Whey protein isolates, and rice and pea protein concentrates. *J. Sci. Food. Agric.* **103**, 7251–7259. 10.1002/jsfa.12809 (2023). 37357639 10.1002/jsfa.12809

[2] Berardy, A., Johnston, C. S., Plukis, A., Vizcaino, M. & Wharton, C. Integrating protein quality and quantity with environmental impacts in life cycle assessment. *Sustainability* **11**, 2747. 10.3390/su11102747 (2019).

[3] Bohn, T. et al. Correlation between in vitro and in vivo data on food digestion. What can we predict with static in vitro digestion models? *Crit. Rev. Food Sci. Nutr.* **58**, 2239–2261. 10.1080/10408398.2017.1315362 (2018). 28613945 10.1080/10408398.2017.1315362

[4] Brodkorb, A. et al. INFOGEST static in vitro simulation of gastrointestinal food digestion. *Nat. Protoc.*10.1038/s41596-018-0119-1 (2019). 30886367 10.1038/s41596-018-0119-1

[5] Brown, E. C., DiSilvestro, R. A., Babaknia, A. & Devor, S. T. Soy versus Whey protein bars: Effects on exercise training impact on lean body mass and antioxidant status. *Nutr. J.* **3**, 22. 10.1186/1475-2891-3-22 (2004). 15588291 10.1186/1475-2891-3-22 PMC539287

[6] Chen, J. et al. A structural explanation for protein digestibility changes in different food matrices. *Food Hydrocoll.* **136**, 108281. 10.1016/j.foodhyd.2022.108281 (2023).

[7] Coluccia, B. et al. Assessing the carbon footprint across the supply chain: Cow milk vs soy drink. *Sci. Total Environ.* **806**, 151200. 10.1016/j.scitotenv.2021.151200 (2022). 34699813 10.1016/j.scitotenv.2021.151200

[8] Egger, L. et al. The harmonized INFOGEST in vitro digestion method: From knowledge to action. *Food Res. Int.* **88**, 217–225. 10.1016/j.foodres.2015.12.006 (2016).

[9] Egger, L. et al. Physiological comparability of the harmonized INFOGEST in vitro digestion method to in vivo pig digestion. *Food Res. Int.* **102**, 567–574. 10.1016/j.foodres.2017.09.047 (2017). 29195987 10.1016/j.foodres.2017.09.047

[10] FAO. Dietary Protein Quality Evaluation in Human Nutrition: Report of an FAO Expert Consultation, 31 March – 2 April 2011, Auckland, New Zealand (FAO, 2013). 26369006

[11] Fernan, C., Schuldt, J. P. & Niederdeppe, J. Health halo effects from product titles and nutrient content claims in the context of protein bars. *Health Commun.* **33**, 1425–1433. 10.1080/10410236.2017.1358240 (2018). 28853950 10.1080/10410236.2017.1358240

[12] Gauza-Włodarczyk, M., Kubisz, L. & Włodarczyk, D. Amino acid composition in determination of collagen origin and assessment of physical factors effects. *Int. J. Biol. Macromol.* **104**, 987–991. 10.1016/j.ijbiomac.2017.07.013 (2017). 28687386 10.1016/j.ijbiomac.2017.07.013

[13] Herreman, L., Nommensen, P., Pennings, B. & Laus, M. C. Comprehensive overview of the quality of plant- and animal-sourced proteins based on the digestible indispensable amino acid score. *Food Sci. Nutr.* **8**, 5379–5391. 10.1002/fsn3.1809 (2020). 33133540 10.1002/fsn3.1809 PMC7590266

[14] Imtiaz, S. R., Kuhn-Sherlock, B. & Campbell, M. Effect of dairy protein blends on texture of high protein bars. *J. Texture Stud.* **43**, 275–286. 10.1111/j.1745-4603.2011.00337.x (2012).

[15] Joint, F. A. O. & WHO/UNU Expert Consultation on Energy and Protein Requirements. *Energy and Protein Requirements: Report of a Joint FAO/WHO/UNU Expert Consultation* (World Health Organization; WHO Publications Center USA [distributor], 1985). 3937340

[16] Loveday, S. M., Hindmarsh, J. P., Creamer, L. K. & Singh, H. Physicochemical changes in a model protein bar during storage. *Food Res. Int.* **42**, 798–806. 10.1016/j.foodres.2009.03.002 (2009).

[17] Małecki, J., Tomasevic, I., Djekic, I. & Sołowiej, B. G. The effect of protein source on the physicochemical, nutritional properties and microstructure of High-Protein bars intended for physically active people. *Foods* **9**, 1467. 10.3390/foods9101467 (2020). 33076297 10.3390/foods9101467 PMC7602487

[18] Mathai, J. K., Liu, Y. & Stein, H. H. Values for digestible indispensable amino acid scores (DIAAS) for some dairy and plant proteins May better describe protein quality than values calculated using the concept for protein digestibility-corrected amino acid scores (PDCAAS). *Br. J. Nutr.* **117**, 490–499. 10.1017/S0007114517000125 (2017). 28382889 10.1017/S0007114517000125

[19] j McMahon, D., l Adams, S. & r McManus, W. Hardening of High-Protein nutrition bars and Sugar/Polyol–Protein phase separation. *J. Food Sci.* **74**, E312–E321. 10.1111/j.1750-3841.2009.01225.x (2009). 19723194 10.1111/j.1750-3841.2009.01225.x

[20] Moughan, P. J. Population protein intakes and food sustainability indices: The metrics matter. *Global Food Secur.* **29**, 100548. 10.1016/j.gfs.2021.100548 (2021).

[21] Moughan, P. J., Butts, C. A., van Wijk, H., Rowan, A. M. & Reynolds, G. W. An acute ileal amino acid digestibility assay is a valid procedure for use in human ileostomates. *J. Nutr.* **135**, 404–409. 10.1093/jn/135.3.404 (2005). 15735070 10.1093/jn/135.3.404

[22] Sousa, R. et al. In vitro digestibility of dietary proteins and in vitro DIAAS analytical workflow based on the INFOGEST static protocol and its validation with in vivo data. *Food Chem.* **404**, 134720. 10.1016/j.foodchem.2022.134720 (2023). 36332577 10.1016/j.foodchem.2022.134720

[23] Regulation (EC) No 1924/2006 of the European Parliament and of the Council of 20 December 2006 on nutrition and health claims made on foods (2006).

